# Altered microRNAs in C3H10T1/2 cells induced by p.E95K mutant IHH signaling

**DOI:** 10.1186/s41065-021-00207-8

**Published:** 2021-12-18

**Authors:** Wei Zhou, Luan Chen, Hao Wu, Ting Wang, Gang Ma, Baocheng Wang, Cong Wang, Na Zhang, Yingtian Zhang, Lin He, Shengying Qin, Xiaofang Sun, Hai Zhang, Lu Shen

**Affiliations:** 1 Department of Obstetrics and Gynecology, Key Laboratory for Major Obstetric Diseases of Guangdong Province, The Third Affiliated Hospital of Guangzhou Medical University, Guangdong Guangzhou, China; 2 Key Laboratory of Reproduction and Genetics of Guangdong Higher Education Institutes, Guangzhou, Guangdong China; 3 Key Laboratory for the Genetics of Developmental and Neuropsychiatric Disorders (Ministry of Education), Bio-X Institutes, Shanghai Jiao Tong University, 200030 Shanghai, China; 4 School of Life Sciences and Biotechnology, Shanghai Jiao Tong University, 200240 Shanghai, China; 5 Institutes of Biomedical Sciences, Fudan University, 200030 Shanghai, China; 6 Xinhua Hospital, Shanghai Jiao Tong University School of Medicine, 200093 Shanghai, China; 7 Department of Pulmonary and Critical Care Medicine, Shanghai Chest Hospital, Shanghai Jiao Tong University, No. 241, West Huaihai Road, 200030 Shanghai, China

**Keywords:** microRNA, Brachydactyly type A1, Indian hedgehog, Pathogenesis

## Abstract

**Background:**

Indian Hedgehog (IHH), an important cell signaling protein, plays a key regulatory role in development of cartilage and chondrogenesis. Earlier studies have shown that heterozygous missense mutations in *IHH* gene may cause brachydactyly type A1 (BDA1), an autosomal dominant inheritance disease characterized by apparent shortness or absence of the middle phalanges of all digits. MicroRNAs (miRNAs) have been found to be significant post-transcriptional regulators of gene expression and significantly influence the process of bone-development. Therefore, it is possible that miRNAs are involved in the mechanism underlying the development of BDA1. However, the relationship between miRNAs and the pathogenesis of BDA1 remains unclear.

**Methods:**

In this study, we used microarray-based miRNA profiling to investigate the role of miRNAs in BDA1 by characterization of differentially expressed miRNAs in C3H10T1/2 cell line induced by wild type (WT) and p.E95K mutant (MT) IHH signaling.

**Results:**

Our results identified 6 differentially expressed miRNAs between WT and control (CT) group and 5 differentially expressed miRNAs between MT and CT groups. In particular, miR-135a-1-3p was found to be a significantly differentially expressed miRNA between WT and CT group. Results of dual-luciferase reporter gene experiment successfully discovered *Hoxd10* was one of the target gene of miR-135a-1-3p. Additionally, our pathway analysis revealed that the targets of these miRNAs of interest were highly involved with Runx1/2, Notch and collagen-related pathways.

**Conclusions:**

Taken together, our findings provided important clue for future study of the process of miRNA-regulation in IHH signaling and novel insights into the regulatory role of miRNA in pathogenesis of BDA1.

**Supplementary Information:**

The online version contains supplementary material available at 10.1186/s41065-021-00207-8.

## Introduction

Hedgehog (HH) signaling pathway is known to be a key regulator in development of cartilage in all the animals that possess bilateral symmetry [1, 2]. In mammals, there are three homologous proteins that are actively involved in the HH signaling pathway: sonic hedgehog (SHH), desert hedgehog (DHH) and Indian Hedgehog (IHH). In particular, IHH signaling pathway is an essential developmental pathway and plays a critical regulatory role in many physiological processes [3]. Members of this pathway are mainly expressed by chondrocytes in mammal development of cartilage [4]. IHH protein interacts with patched 1 (PTCH1), a transmembrane receptor protein of its receptor. The interaction between IHH and PTCH1 results in the suppression of smoothened (SMO). The suppression of SMO allows transducing activation signal to the downstream glioma-associated oncogene homolog (Gli) transcription factors [5, 6]. IHH/Gli signaling pathway induces differentiation of osteoblasts and plays an important role in endochondral osteogenesis [7]. Any abnormalities in IHH/Gli signaling pathway leads to abnormal bone development.

MicroRNAs (miRNAs) are single-stranded non-coding RNAs (17-25nt) that are known to play a key role in various cellular pathways, including pathways of growth and proliferation, apoptosis, and developmental timing [8–10]. MiRNAs are significant post-transcriptional regulators of gene expression that have been founded in a great number of organisms [11, 12]. MiRNAs negatively regulate specific messager RNA (mRNA) translation by direct binding to partially complementary sequences in target mRNA. Previous studies have shown that miRNAs negatively regulate osteoblast differentiation and bone formation by targeting related signaling pathways or downstream transcription factors [13, 14]. For example, miRNA-467g has been found to inhibit new bone regeneration through suppression of HH signaling pathway by targeting Runx-2[13]. However, the mechanism of miRNA-mediated regulation of abnormalities in IHH pathway remains unclear.

Brachydactyly type A1 (BDA1) is an autosomal dominant inheritance disease caused by missense mutations of heterozygotes in IHH. It is a dysplasia/aplastic disorder characterized by the shortening or missing of the middle phalanges [15, 16]. A number of earlier studies have suggested that p.E95K in the N-terminal fragment of IHH protein, a mutation found in BDA1 individuals, affected the interaction between PTCH1 and its partners. This interaction of PTCH1 may be able to prevent the induction of the signaling [17]. The p.E95K mutation has been found to affect the differentiation of chondrocyte and severely impair the development process of cartilage, resulting in a delayed formation of endochondral bone and brachydactyly phenotype [6]. It has also been found that the p.E95K mutant IHH protein affected Gli-mediated downstream regulation through a series of signal transduction [18]. However, the modulation of miRNAs in p.E95K mutant IHH signaling remains unknown.

In this study, we performed a microarray-based screening of miRNA expression in response to wild type (WT) or p.E95K mutant (MT) IHH signaling to identify the differentially expressed miRNAs. Our results will help to explain the mediation role of miRNA in the regulation in IHH signaling and provide novel insight into the molecular mechanisms underlying p.E95K IHH induced BDA1.

## Materials and methods

### IHH protein expression and purification

The pGEX-2T-based recombinant human IHH-N-terminus proteins (IhhN), including wild type protein (WT, amino acid residues 28-202) and a mutant protein (WT, p.E95k), were expressed by the BL21 (DE3) strain of *Escherichia coli* (*E. coli*). We obtained the two transformed strains from Bio-X Institutes, Shanghai Jiao Tong University, which had been described in earlier publication [19]. After coated plates, monoclonal colony was selected and incubated in 5ml LB liquid medium with ampicillin at 37℃ overnight. Then the medium was transferred to 400 ml 2×YT liquid medium with ampicillin and incubated at 37℃ for 4 h. When the value of OD600 reached 0.6, the strains were induced with 0.2 mM IPTG at 32℃ for 5 h. Pellets were collected by centrifugation at 8 000× g for 10 min at 4℃ and re-suspended in 200 mM NaCl and 20 mM Tris-HCl, pH 7.5 with cocktail protein inhibitors (Sigma-Aldrich), followed by sonication. After centrifugation at 25,000× g for 30 min at 4℃, the supernatant was collected. Then the proteins were purified according to the Bulk and RediPack GST Purification Modules (GE-Healthcare), followed by cleavage of the GST tag using thrombin. The purified IhhN proteins were verified by western blotting with IHH Antibody (SC-1782, Santa Cruz) and filtered by 0.22 μm filter (Millipore).

### IHH activation of C3H10T1/2 cells

The induction was performed using the method that had been described in previous study [19]. C3H10T1/2 cells (obtained from ATCC) were chosen to analyze IHH signaling in the process of osteoblast differentiation of the mesenchymal cell line [20]. C3H10T1/2 cells were cultured in 12-well plates in a growth medium containing MEM/EBSS, 10 % fetal bovine serum (FBS), and 2 % penicillin/streptomycin. WT or MT IhhN (E95K) was added into the growth mediums (1x = 750 nM IhhN protein finally) when cells grew to 5 × 10^6^ per well. After 48 h of further incubation at 37℃ (5 % CO2), the cells were harvested for further analysis. Expression of *Gli1*, *PTCH1* and *IHH*, as markers of IHH signaling pathway, were detected by real time PCR. All the induction assays were performed in triplicate.

### Microarray-based miRNA Expression Analysis

We used TRIZOL™ solution (Invitrogen) to extract the total RNA from cell samples according to the manufacturer’s instruction. RNAs were examined by Agilent 2100 Bioanalyzer to assure its RIN to be more than 7. MicroRNA microarray analyses were performed using 200 ng total RNA on Unrestricted Mouse miRNA Microarray v2 (Agilent Technologies). The amplification, labeling, hybridization and scanning of samples were performed by Agilent Technologies (Shanghai, China) using standard protocols. The data was extracted by Feature Extraction version 12.5 (Agilent Technologies) and analyzed by Genespring GX version 12.6 (Agilent Technologies). The raw data can be obtained from GEO (GSE74023).

### Bioinformatics Analysis

Target genes regulated by differentially expressed miRNAs (DEMs) were predicted using the TargetScan database [21] (Version 7.2) and DIANA-microT database [22, 23]. The top 5 % predicted targets of each DEM in these two datasets were obtained and their overlapped genes were selected. Pathway analysis was conducted by Kyoto Encyclopedia of Genes and Genomes (KEGG) database [24–26] and Reactome database [27]. Rnacentral database [28] was used to explore the literatures that were relevant to miR135a-1-3p. All figures and statistics method were performed by R 4.0.

### Validation target gene of mir135a by dual-luciferase report experiment

293T cells were plated onto the 48-well plates at 37 °C and cultured to reach 70 %-80 % confluence. Fragment of the 3’UTR of *Hoxd10* was inserted into the psiCHECK-2 Luciferase vector (Promega, Madison, Wisconsin, USA). The designed mmu-miR-135a-1-3p mimics and NC mimics was used to co-transfect 293T cells together with the corresponding plasmids (Wide type *Hoxd10*) using Lipofectamine 2000 for 48 h. For assessment of action of mmu-miR-135a-1-3p on *Hoxd10*, cells were grouped as following: (A) NC group, cells co-transfected with WT *Hoxd10* plasmid and NC mimics; (B) mmu-miR-135a-1-3p mimics, cells co‐transfected with WT *Hoxd10* plasmid and mmu-miR-135a-1-3p mimics group. The luciferase report activity was measured using Dual Luciferase reporter 1000 Assay System (Promega). The data was normalized by the rellina luciferase activity of control group. Each experiment was performed in triplicate.

## Results

### Differentially expressed miRNAs in activated IHH signaling pathway

**Fig. 1:**
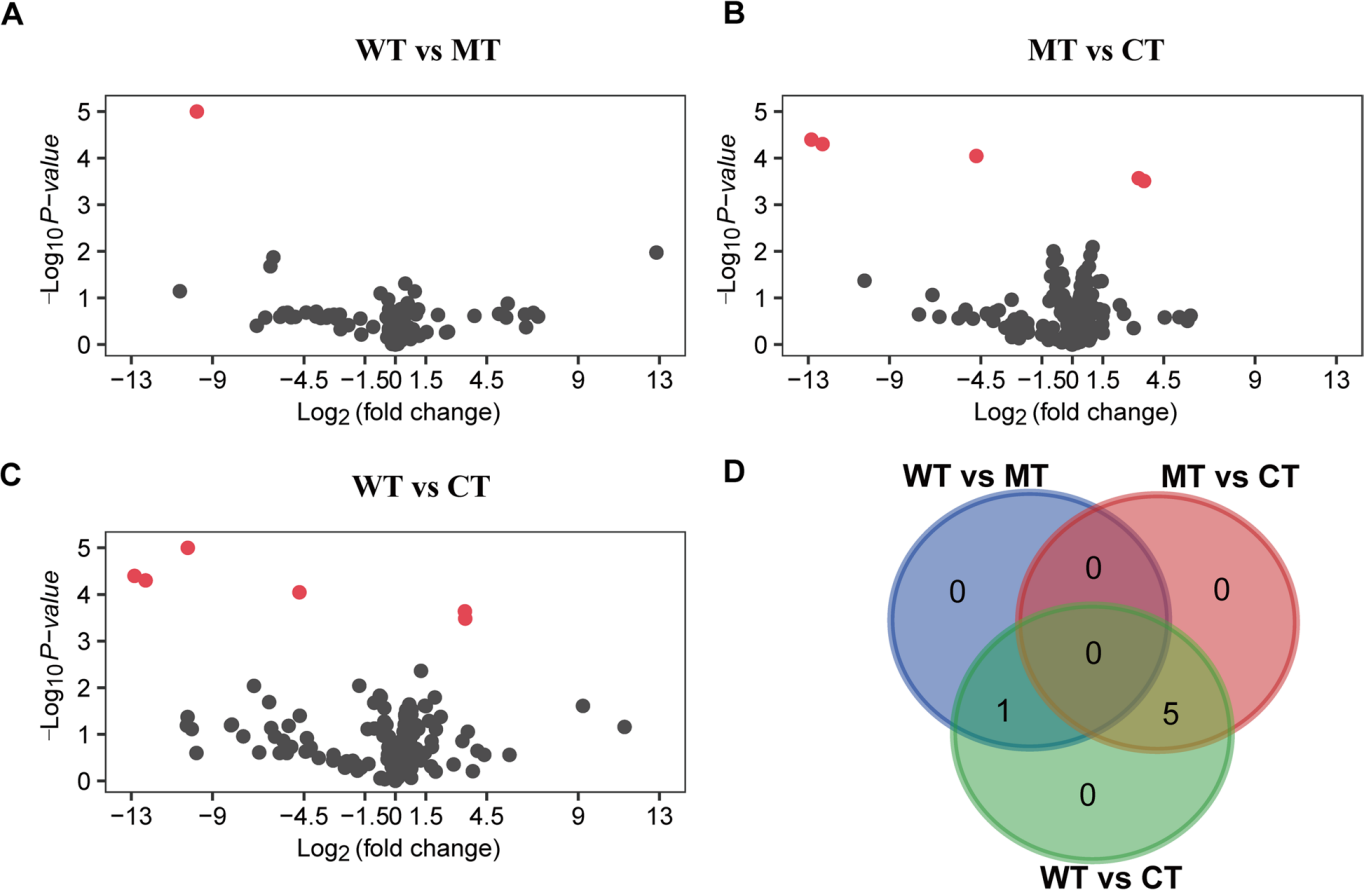
Volcano plot for Differentially expressed miRNAs in activated IHH signaling pathway. **A** The differentially expressed miRNAs between WT an MT group; **B** The differentially expressed miRNAs between MT an CT group; **C** The differentially expressed miRNAs between WT an CT group; **D** venn chart for the three group. WT: wild type group, MT: p.E95K mutant group, CT: control group. Red dot means the differentially expressed miRNAs

Cells were divided into WT, MT and CT groups according to their treatments (activated by WT IhhN, activated by MT IhhN and unactivated respectively). There were two biological replicates within each group. 650 probe features were derived from the microarray. Among them, we selected 169 probe features that had gene information to perform further analysis. The difference between the spots and the median of the same spot across all the arrays were evaluated by the relative log expression (RLE), which was computed for every spot in the array. The majority of the spots was centered around zero, indicating that most of the miRNAs among three groups were not differentially expressed. A few miRNAs were significantly differentially expressed across the three groups (Fig. 1, Additional file 1, 2, 3 and 4). As shown in Table 1, there were 6 DEMs between WT and CT group; 5 DEMs between MT and CT groups (*P*-value < 0.05). In particular, miR-135a-1-3p was the only DEM between WT and MT group (Table 1).

**Table 1:** Differentially Expressed miRNAs

CONTRAST	WT vs CT	MT vs CT	WT vs MT
All DEMs	6	5	1
UP	mmu-miR-29b-3p	mmu-miR-29b-3p	NULL
	mmu-miR-30e-5p	mmu-miR-30e-5p	
DOWN	mmu-miR-135a-1-3p	mmu-let-7c-1-3p	mmu-miR-135a-1-3p
	mmu-let-7c-1-3p	mmu-miR-101a-3p	
	mmu-miR-101a-3p	mmu-miR-712-5p	
	mmu-miR-712-5p		

## Predicted targets of DEMs

**Fig. 2:**
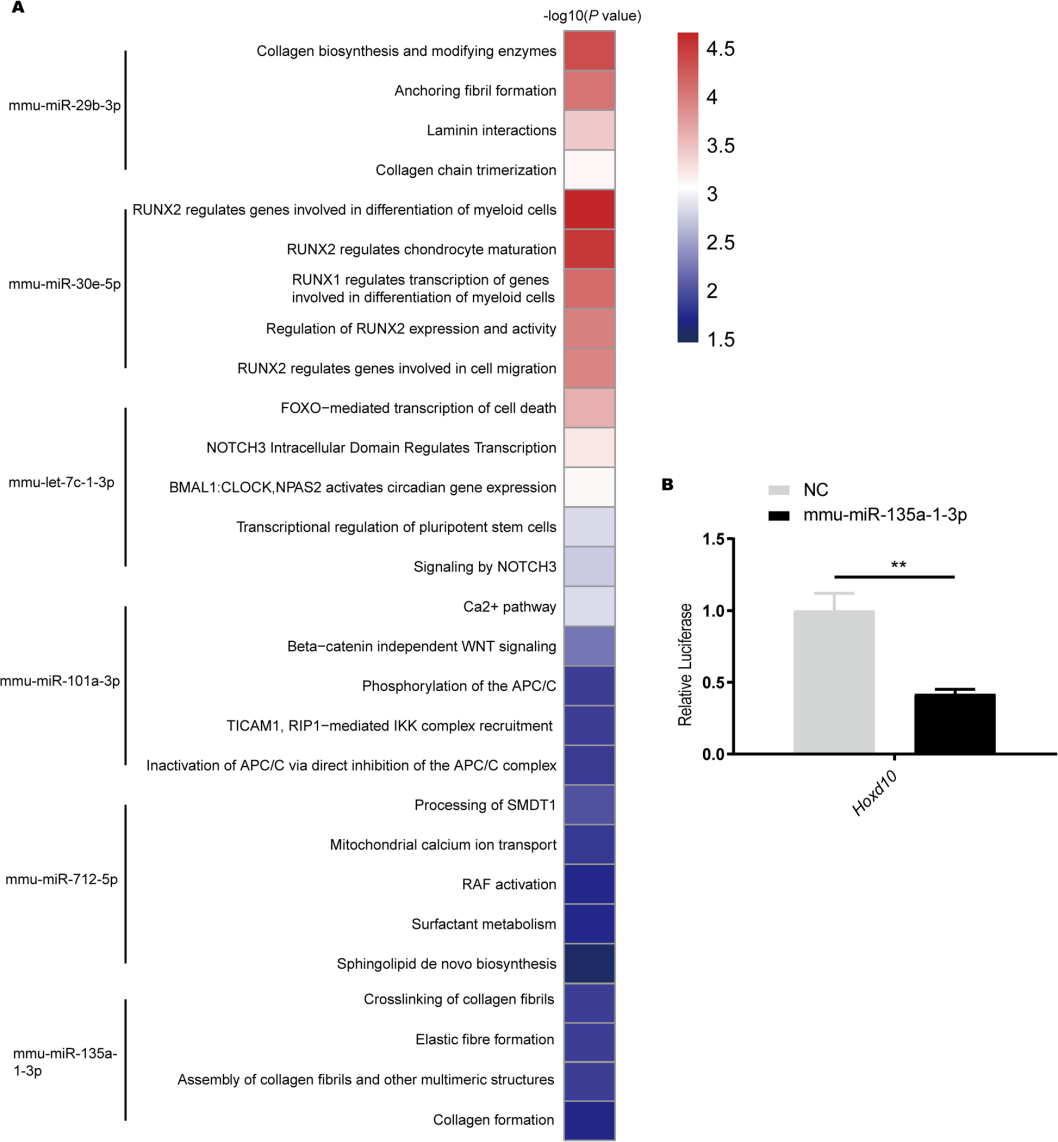
Heatmap and the result of dual-luciferase report experiment. **A** Heatmap for enrichment of the differentially expressed miRNAs targets. **B** The dual-luciferase reporter gene experiment for miR135-1-3p and its target *Hoxd10*. **P < 0.01, analyzed by *t* test

The top 5 % predicted targets of each DEMs using TargetScan database [21] (Version 7.2) and DIANA-microT database [22, 23] were obtained. The common predicted genes from two datasets are shown in Additional file 5. Pathway analyses were conducted based on Kyoto Encyclopedia of Genes and Genomes (KEGG) database [24–26] and Reactome database [27] to find the enrichment of DEM targets. The top 5 most significantly related pathways are shown in Fig. 2 A and Supplementary files (Additional files 6, 7 and 8). Runx1/2-related pathways were significantly enriched in the targets of up-regulated miRNAs (4 of the top 5 pathways, Additional file 6). Notch3 signaling pathways were significantly enriched in the targets of down-regulated miRNAs (2 of the top 5 pathways, Additional file 7). Runx1/2 and collagen-related pathways were enriched in the targets of all DEMs (Additional file 8). Notch signaling pathway was enriched in the targets of miR135-1-3p (Additional file 9). These pathways mentioned above had been reported to be closely related to chondrogenesis [29, 30].

### MiR135-1-3p regulates the expression of *Hoxd10* in 293T cells

In this step, we validated the relationship between miR135-1-3p and *Hoxd10* to confirm if miR135-1-3p regulated the process of endochondral osteogenesis. *Hoxd10* is one of the predicted target gene of miR135-1-3p. *Hoxd10* has been reported to be expressed in the developing limb buds and was involved in differentiation and limb development [31]. The dual-luciferase reporter gene experiment was performed to detect if miR135-1-3p directly combined with the 3′-UTR of the *Hoxd10* mRNA. Compared to the control group (Mir-NC), the luciferase activity of the *Hoxd10* of the miR-135a-1-3p group was significantly lower (Fig. 2B, *P* = 0.0014).

### Validation of potential targets of DEMs by gene expression profiles

Expressions of predicted targets were examined based on mRNA expression profiles that had been described in our previous study [19]. We successfully identified 9 target genes (Table 2). MiRNAs silence the target genes by base pairing with complementary sequences within mRNA molecules. Therefore, we considered a pair of a miRNA and its regulation target were a gene and a DEM with opposite expression states. Our result showed that miR-29-3p/*Col4a5*, miR-29-3p/*Dpysl5*, miR-712-5p/*Gpr116* and miR-101a-3p/*Nova1* were highly confident miRNA-gene pairs (Absolute fold change cutoff = 2). However, the function of these genes did not appear to be related to endochondral osteogenesis.

**Table 2:** Pairs of DEMs and their predicted target genes

Gene	mRNA	Expression in MT group	Expression in WT group	Targeted by DEM (mouse)	Up/Down Regulation of DEM
		(Fold change, vs Control)	(Fold change, vs Control)		(vs Control)
*Chn1*	NM_029716	-2.78	-1.31	miR-712-5p	DOWN
*Col4a5*	NM_007736	-1.28	-2.49	miR-29-3p	UP
*Dpysl5*	NM_023047	-3.72	-3.2	miR-29-3p	UP
*Gpr116*	NM_001081178	1.07	2.22	miR-712-5p	DOWN
*Gpr37*	NM_134438	1.79	2.48	miR-29-3p	UP
*Lin28b*	NM_001031772	3.61	4.26	miR-30e-5p	UP
*Mycn*	NM_008709	1.24	1.65	miR-101a-3p	DOWN
*Nova1*	AK034178	2.51	1.09	miR-101a-3p	DOWN
*Slc25a12*	AK086488	1.97	1.99	let-7c-1-3p	DOWN

## Discussion

Previous studies have revealed that the stability of E95K mutant IHH and wild type IHH protein were similar [17]. However, a recent study has reported a different conclusion, suggesting that the mutant IHH impaired its interactions with its partners in mouse model with digit abnormalities [6]. In addition, several studies on molecular mechanism of downstream regulation involving the mutant IHH signaling have been published. To our knowledge, this study is the first attempt to characterize the miRNA-mediated regulation in BDA1-related mutant IHH signaling and demonstrated that a number of chondrogenesis-relevant pathways, including Runx1/2-related, Notch3 signaling and collagen-related pathways, were significantly enriched in the target genes of DEMs. Our results suggested that miRNA regulation in IHH signaling might play a crucial role in chondrogenesis.

The result of differential miRNA profiling showed that Runx1/2-related pathways were enriched in the DEMs. Earlier studies have shown that Runx1 contributed to parathyroid hormone (PTH)-induced chondrogenesis and the induction of mesenchymal stem cell commitment to the early stages of chondrogenesis [29]. The knockdown of Runx1 completely blunted PTH-mediated chondrogenesis [29, 32]. IHH stimulates the expression of PTH-related peptide (PTHrP), a protein that negatively regulates terminal chondrocyte differentiation through the PTH/PTHrP receptor (PPR) [33]. Moreover, it has been reported that the duplicated copy number of *PTHrP* caused BDA1 phenotype [34]. Recent studies have found that the copy number variation of *RUNX2* resulted in brachydactyly-liked phenotypes [35–37]. Taking together, our results indicated that the identified DEMs might be responsible for the pathogenesis of BDA1 by exerting influence on the regulation of Runx1/2.

Our study identified a miRNA (miR-135a-1-3p) had the significant difference in expression level between WT and MT group. The dual-luciferase reporter gene experiment verified that *Hoxd10* was a direct target of miR-135a-1-3p, who may suppress the expression of *Hoxd10*. *Hoxd10* encodes a sequence-specific transcription factor, which has been found to be expressed during the developing of limb buds. *Hoxd10* is critical in limb formation and limb morphogenesis [38]. Carpenter’s team has reported that targeted disruption of *Hoxd10* gene caused hindlimb-specific defects in gait and adduction in mice [39]. Hence, miR-135a-1-3p may regulate the IHH signaling by target *Hoxd10.*

The results of pathway analysis revealed that collagen fibrillogenesis-related pathways were enriched in the target genes of miR135-1-3p. Collagens have been recognized as candidate indicators of the cartilage phenotype in process of chondrogenesis and chondrocyte differentiation [40]. PTHrP/Ihh axis has been found to control the expression of collagen VI by proliferating chondrocytes during the development of limb [41]. Therefore, miR-135a-1-3p may be associated with the pathogenesis of BDA1 and chondrogenesis. Furthermore, it is noteworthy that the expression levels of mmu-let-7c-1-3p in both WT and MT group were lower than the one in CT group. However, the expression levels of mmu-let-7c-1-3p in WT and MT group appeared to have no difference. NOTCH3 signaling pathway was the most significant pathway enriched in target genes of mmu-let-7c-1-3p. It has been reported that Notch signaling plays an important role in the coordination of perichondrial osteoblast differentiation, regulation of the communication between chondrocytes and perichondrial osteoblasts and promotion of chondrocyte proliferation and apoptosis. Further, the inhibitory roles of Indian hedgehog (Ihh) downregulation on chondrocyte growth and differentiation [42]. Therefore, our findings indicated that mmu-let-7c-1-3p may be regulate the IHH signaling pathway to inhibit chondrocyte differentiation.

## Conclusions

This is the first study attempted to reveal the role of miRNA regulation involved in the pathogenesis of BDA1. Six miRNAs were found to have significant difference in expression level between induced and control group. We discovered that miR-135a-1-3p was a significantly differentially expressed miRNA. *Hoxd10* was identified as a target of miR-135a-1-3p, who is involved in chondrogenesis. Pathway analysis revealed that Runx1/2, Notch and collagen-related pathways were enriched in the top-confident predicted targets of these six miRNAs, which were closely related to chondrogenesis. Our results indicated the important role of miRNA-regulation in the pathogenesis of BDA1 and chondrogenesis process.

## Supplementary Information

**Additional file 1.** Mean signal of microarrays.

**Additional file 2.** M-A plot of WT vs CT.

**Additional file 3.** M-A plot of MT vs CT.

**Additional file 4.** M-A plot of WT vs MT.

**Additional file 5.** Pathway analysis of targets of all DEMs.

**Additional file 6.** Pathway analysis of targets of all up-regulated DEMs.

**Additional file 7.** Pathway analysis of targets of all down-regulated DEMs.

**Additional file 8.** Pathway analysis of targets of all DEMs.

**Additional file 9.** Pathway analysis of targets of miR-135a-1-3p.

## Data Availability

All requests for raw and analyzed data will be reviewed by the corresponding author. The detailed information of the primer is shown in the supplementary data.

## Abbreviations

IHH: Indian Hedgehog
BDA1: brachydactyly type A1
miRNAs: microRNAs
WT: wild type
MT: p.E95K mutant
HH: Hedgehog
SHH: sonic hedgehog
DHH: desert hedgehog
mRNA: messagerRNA
DEM: differentially expressed miRNAs
IhhN: IHH-N-terminus proteins
FBS: fetal bovine serum
KEGG: Kyoto Encyclopedia of Genes and Genomes
MIQE: Minimum Information for Publication of Quantitative Real-Time PCR Experiment
CT: control
RLE: relative log expression
PTH: parathyroid hormone
PTHrP: PTH-related peptide
