# Supplementary figures and images for: Altered microRNAs in C3H10T1/2 cells induced by p.E95K mutant IHH signaling
Source: Hereditas. 2021 Dec 18;158:48. doi: 10.1186/s41065-021-00207-8 (PMC8684136; doi:10.1186/s41065-021-00207-8)

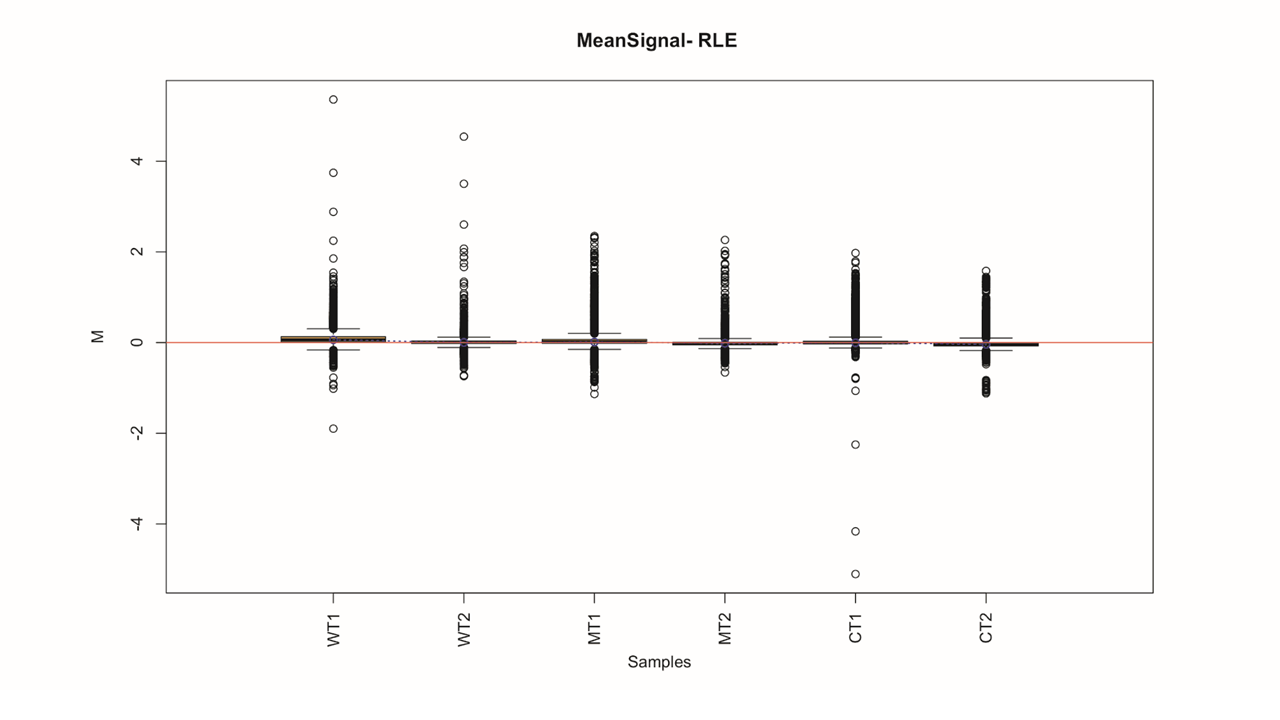

Supplement: Supplementary file 1 — Additional file 1. Mean signal of microarrays. [file 41065_2021_207_MOESM1_ESM.tif]

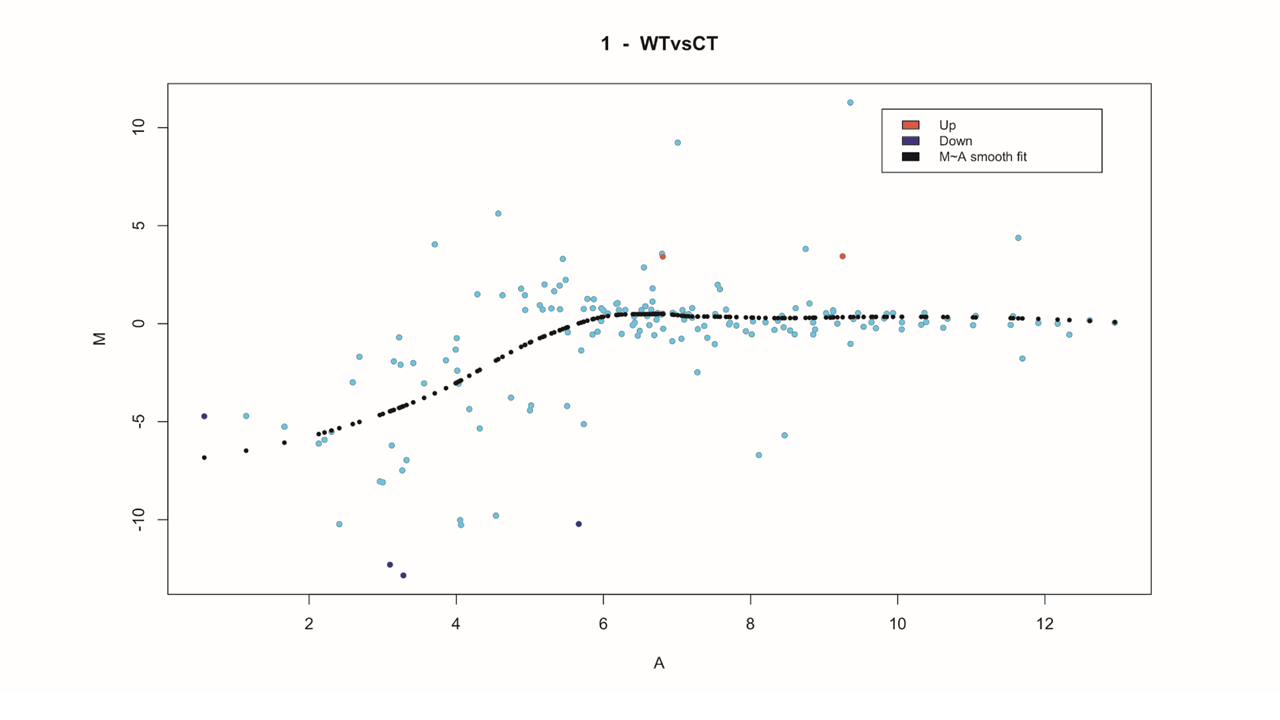

Supplement: Supplementary file 2 — Additional file 2. M-A plot of WT vs CT. [file 41065_2021_207_MOESM2_ESM.tif]

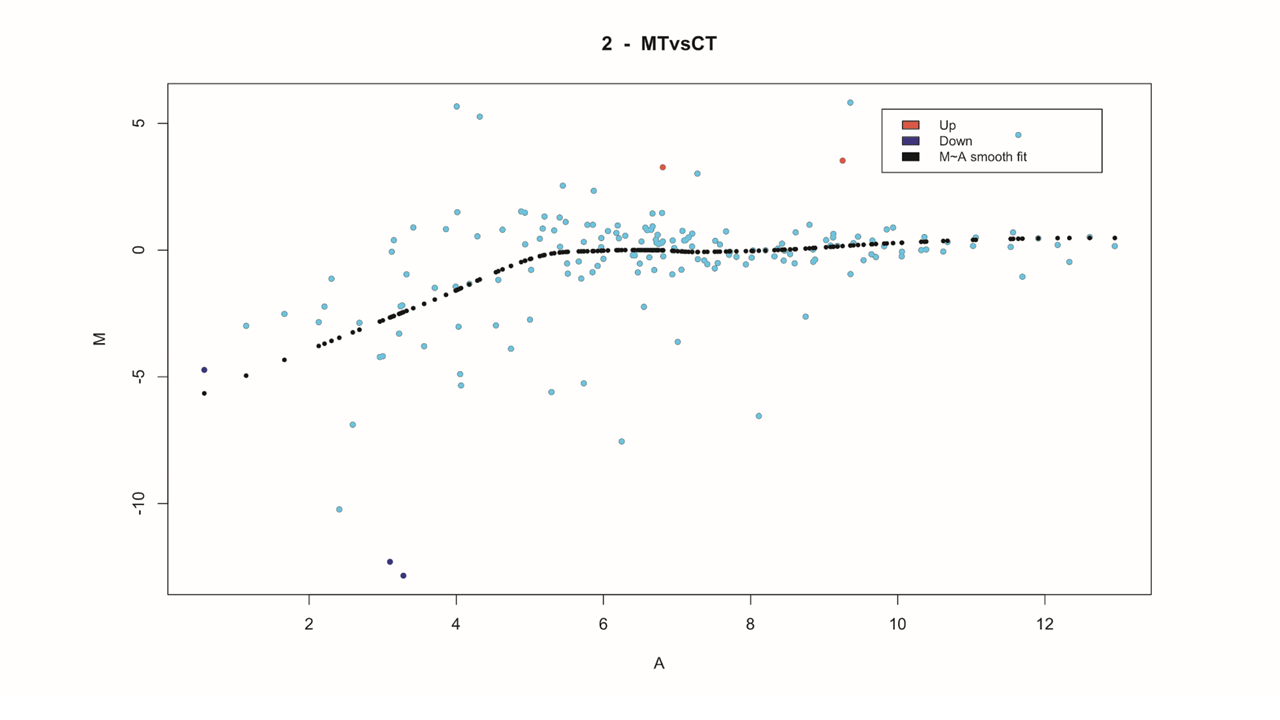

Supplement: Supplementary file 3 — Additional file 3. M-A plot of MT vs CT. [file 41065_2021_207_MOESM3_ESM.tif]

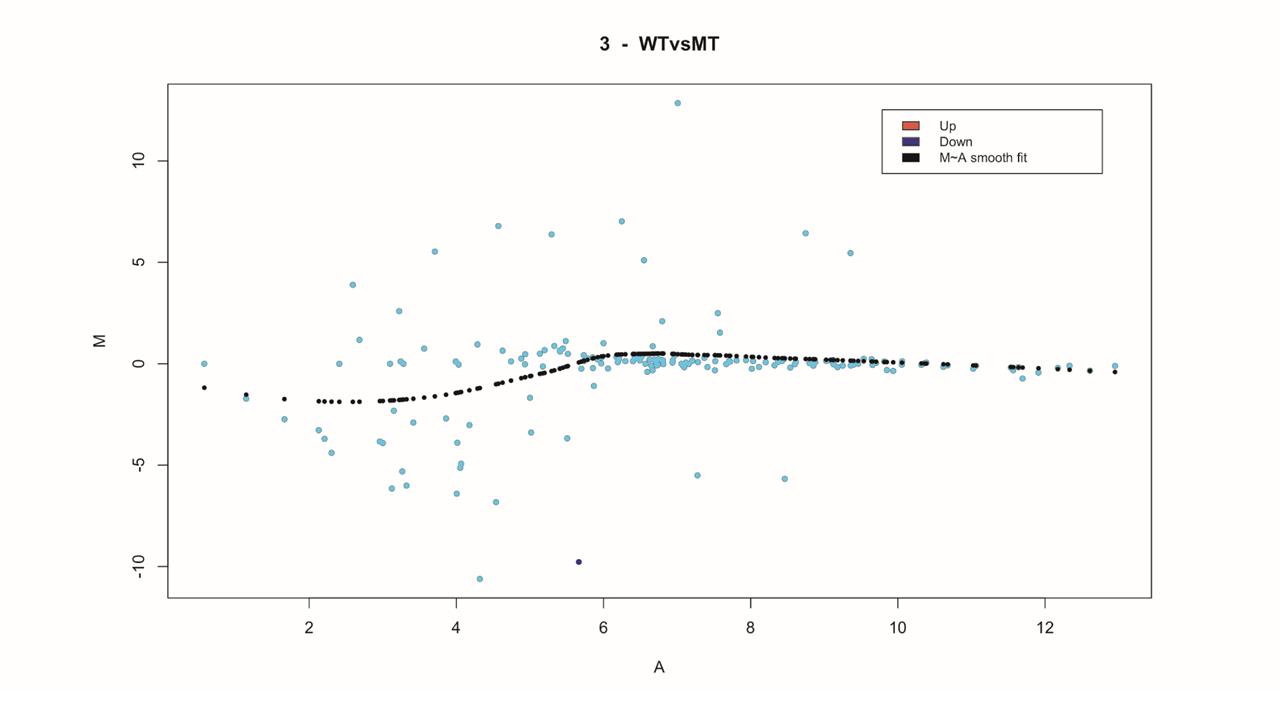

Supplement: Supplementary file 4 — Additional file 4. M-A plot of WT vs MT. [file 41065_2021_207_MOESM4_ESM.tif]
